# Benzoxazine Monomers and Polymers Based on 3,3′-Dichloro-4,4′-Diaminodiphenylmethane: Synthesis and Characterization

**DOI:** 10.3390/polym13091421

**Published:** 2021-04-28

**Authors:** Viktoria V. Petrakova, Vyacheslav V. Kireev, Denis V. Onuchin, Igor A. Sarychev, Vyacheslav V. Shutov, Anastasia A. Kuzmich, Natalia V. Bornosuz, Mikhail V. Gorlov, Nikolay V. Pavlov, Alexey V. Shapagin, Ramil R. Khasbiullin, Igor S. Sirotin

**Affiliations:** 1 Faculty of Petroleum Chemistry and Polymeric Materials, Mendeleev University of Chemical Technology of Russia, Miusskaya Sq. 9, 125047 Moscow, Russia; petrakovavv@muctr.ru (V.V.P.); kireev@muctr.ru (V.V.K.); donuchin@muctr.ru (D.V.O.); yahoo123-92@mail.ru (I.A.S.); shutov1105@gmail.com (V.V.S.); akuzmich@muctr.ru (A.A.K.); bornosuz@muctr.ru (N.V.B.); mgorlov@muctr.ru (M.V.G.); npavlov@muctr.ru (N.V.P.); 2 All-Russian Scientific Research Institute of Aviation Materials, 105275 Moscow, Russia; 3 Frumkin Institute of Physical Chemistry and Electrochemistry, Russian Academy of Sciences (IPCE RAS), 119071 Moscow, Russia; shapagin@mail.ru (A.V.S.); khasbiullin@techno-poisk.ru (R.R.K.)

**Keywords:** benzoxazines, polybenzoxazines, diaminodiphenylmethane, 3,3′-dichloro-4,4′-diaminodiphenylmethane, heterocycles, thermosetting binders

## Abstract

To reveal the effect of chlorine substituents in the ring of aromatic amine on the synthesis process of benzoxazine monomer and on its polymerization ability, as well as to develop a fire-resistant material, a previously unreported benzoxazine monomer based on 3,3′-dichloro-4,4′-diaminodiphenylmethane was obtained in toluene and mixture toluene/isopropanol. The resulting benzoxazine monomers were thermally cured for 2 h at 180 °C, 4 h at 200 °C, 2 h at 220 °C. A comparison between the rheological, thermal and fire-resistant properties of the benzoxazines based on 3,3′-dichloro-4,4′-diaminodiphenylmethane and, for reference, 4,4′-diaminodimethylmethane was made. The effect of the reaction medium on the structure of the oligomeric fraction and the overall yield of the main product were studied and the toluene/ethanol mixture was found to provide the best conditions; however, in contrast to most known diamine-based benzoxazines, synthesis in the pure toluene is also possible. The synthesized monomers can be used as thermo- and fire-resistant binders for polymer composite materials, as well as hardeners for epoxy resins. Chlorine-containing polybenzoxazines require more severe conditions for polymerization but have better fire resistance.

## 1. Introduction

Phenol-formaldehyde (phenolic) resins are widely used in construction [1], electronics [2] and the aerospace sector [3] because of their low cost and relatively high performance, for example, in heat and fire resistance [4]. However, phenolic resins have a number of disadvantages that limit their use: the release of volatile substances during curing and the formation of micropores inside the material, which impair the properties of the final products or require autoclave processing to obtain high-performance material [5]. Benzoxazines are a novel analogue of phenolic resins with improved characteristics, mainly due to polymerization without the release of volatile substances and wide possibilities of copolymerization with thermosets of other classes. Polybenzoxazines are characterized by a number of advanced properties, including high mechanicals, dimensional stability and heat resistance, near-zero curing shrinkage, and low moisture absorption [6,7]. The combination of these properties have attracted a lot of attention from materials scientists, and many resin and prepreg manufacturers have developed and commercialized benzoxazine-based products for composites, coatings and the electronics industry [8,9]. Bifunctional monomers based on various architectures built up from diamines and monofunctional phenols seem to be more perspective compared to those based on diphenols and monoamines, since they are characterized by enhanced mechanical properties, thermal and fire resistance [5,10,11,12,13,14,15].

However, until recently, the synthesis of benzoxazine monomers based on diamines and monophenols was practically impossible. At the first stage of the condensation reaction hyperbranched crosslinked structures based on diamine and paraformaldehyde are formed, which can precipitate from the reaction mixture, thereby reducing the main product yield [16]. This circumstance excluded the possibility of carrying out a one-stage synthesis until it was shown that the proper selection of synthesis conditions allows researchers to obtain bifunctional monomers based on diamines and phenols [14]. In a number of recent works [14,17,18,19,20,21], it was shown that in certain conditions, namely, carrying out the reaction in a solvent mixture of toluene/ethanol (2:1), it is possible to obtain the target product in one stage.

At temperatures above 180 °C, these monomers undergo thermal polymerization via the ring-opening of the oxazine cycle without the release of low-molecular byproducts. This process is accompanied by the formation of crosslinked polymers, and so the monomers can be used as binders for composite materials, both as a separate components of a thermosetting system, and as a co-monomers in systems with epoxy resins [22], bismaleimides, polyurethanes, cyan ethers, phthalonitriles, etc. to form highly crosslinked copolymers [23]. Benzoxazine monomers can be processed by resin transfer molding (RTM) and vacuum infusion due to the wide processing window [24,25,26,27].

The modern composite industry requires a variety of components for binders, for example, to obtain structural materials with reduced flammability and heat resistance. Thus, the search for new benzoxazine monomers with improved properties is an urgent task.

We have chosen 3,3′-dichloro-4,4′-diaminodiphenylmethane (quamin, a chlorinated analog of the well-known industrial diamine 4,4′-diaminodiphenylmethane) as the starting reagent for the synthesis of the P-d benzoxazine monomer. It is believed that the addition of chlorine atoms to the monomer structure will improve the fire and heat resistance properties of the materials [28,29,30,31]. An additional task was to reveal the effect of the chlorine substituents in the aromatic ring of the amine on the synthesis process of benzoxazine monomer and on its ability to undergo polymerization. The lower activity of quamin in condensation reactions with formaldehyde [32] allows us to make an assumption that special conditions (a mixture of toluene and ethanol solvents) are not necessary in this case. Electron-withdrawing chlorine atoms can also hinder the polymerization of benzoxazines [33]. These assumptions required experimental confirmation, which was the aim of this article. As a reference monomer, we used P-d based on 4,4′-diaminodiphenylmethane. The second component for both diamines was phenol. The thermal and rheological characteristics of both diamine-based benzoxazines were investigated within this work.

## 2. Materials and Methods

### 2.1. Starting Materials

4,4′-diaminodiphenylmethane (d) 97% (Alfa Aesar, Kandel, Germany), 4,4′-diamino-3,3′-dichlorodiphenylmethane (quamin or q) 97% (Chimex Limited, Saint Petersburg, Russia), phenol purified by distillation, paraformaldehyde 91% (ERCROS, Barselona, Spain) used without cleaning; toluene, isopropanol (Komponent-Reaktiv, Moscow, Russia) were dried using molecular sieves and distilled before use.

### 2.2. Synthesis of Benzoxazine Monomers Based on Diamines of Various Structures

The calculated amount of diamine, phenol and solvent was loaded (Table 1) into a 500 mL round-bottom flask equipped with a magnetic stirrer and a reflux condenser. The dissolution of solid reagents was carried out at 60 °C, then the calculated amount of paraformaldehyde was loaded. The temperature was raised to 80–90 °C and the synthesis was carried out for 8 h. Then, the solvents were removed and the product was dried at 90 °C in a vacuum oven for 6 h. The benzoxazine monomers were obtained as yellow glassy substances with a softening point temperature range of 80–100 °C in a 90–95% yield. Resulting monomers were used for further polybenzoxazines synthesis without additional purification.

### 2.3. Curing of Benzoxazines

Benzoxazine monomers were cured according to the following regime: 2 h at 180 °C, 4 h at 200 °C, 2 h at 220 °C; all samples were degassed at 130 °C for 1 h before curing. The completeness of the curing process was controlled by the absence of an exothermic effect on the DSC thermogram.

### 2.4. Measurements

^1^H, ^13^C NMR spectra were measured in a CDCl_3_ solution using a Bruker AV-600 spectrometer (Bruker Corporation, Bremen, Germany) operating at frequencies of 600 and 162 MHz, respectively. Chemical shifts are reported in parts per million and referenced to the signals of deuterated solvents. ^1^H NMR chemical shifts are reported relative to the signals of tetramethylsilane. The spectra were processed using the MestReNova Lab package (version 12.0.4, MESTRELAB RESEARCH, S.L., Santiago de Compostela, Spain).

Differential scanning calorimetry (DSC) was measured on a Netzsch DSC 204 F1 Phoenix instrument (Netzsch, Selb, Germany) in a nitrogen atmosphere (50 mL/min) at a heating rate of 10 °C/min.

Thermogravimetric analysis (TGA) with quadrupole mass spectrometry (QMS) were carried out on a Netzsch TG 209 F1 Iris (Netzsch, Selb, Germany) and QMS 403 D Aeolos (Netzsch, Selb, Germany), respectively. TGA was carried out at a heating rate of 20 °C/min and an inert atmosphere flow rate of 50 mL/min. The temperature of transport capillary was 230 °C. The curves were processed using the Netzsch Proteus Thermal Analysis version 6.1.0 (Netzsch, Selb, Germany). QMS was carried out in analog scan mode on the detector CH-TRON and in mass mode SCAN-F. The detector voltage was 1200 V. The holding time for the measurement was 0.5, 1 and 2 s. The curves were processed using the Inficon AG QUADSTAR v7.02 (Netzsch, Selb, Germany).

Flammability tests were carried out in accordance with Vertical Burning Test UL-94 (ASTM D3801–20a, Northbrook, IL, USA) [34] on 5 samples. The dimensions of the samples were 127 mm × 12.7 mm × 2 mm.

Studies of rheological properties were carried out on the Anton Paar Modular Rheometer MCR 302 (Graz, Austria) in oscillation mode at frequency of 1 Hz, shear strain of 10% and a gap 0.5 mm using plate-plate geometry.

FTIR spectra were recorded on a Nicolet 380 Fourier spectrophotometer with an ATR accessory.

The morphology and the char yield of the P-q-based polymer samples were investigated by scanning electron microscopy on a JSM-6510 device (JEOL, Tokyo, Japan) at an accelerating voltage of 15 kV.

SEM images and FTIR spectra were obtained with the use of char samples after ignition during vertical test according to UL-94 standard.

Elemental analyzer for sulfur, chlorine, nitrogen and carbon Multi EA 5000 (Analytik Jena AG, Jena, Germany) was used.

Investigation of elemental composition of samples was carried out on scanning-electron microscope (Jeol JSM-U3, Tokyo, Japan) with energy dispersive X-ray spectrometer (Eumex, Heidenrod, Germany) in accelerating voltage of 15 kV. Chemical elements were identified by the characteristic Kα-lines of the X-ray spectrum.

## 3. Results and Discussion

### 3.1. Synthesis of Benzoxazine Monomers Based on Diamines

**Figure 1:**
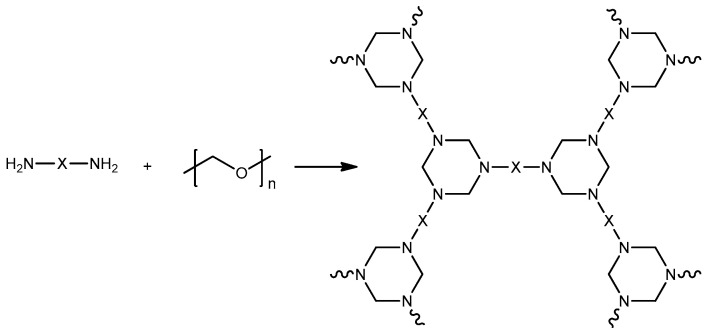
Possible side reaction during the production of benzoxazine monomers based on diamines.

The preparation of benzoxazines based on di- and polyamines is complicated by the peculiarity of the first stage of the reaction. The formation of hyperbranched triazine chains (Figure 1), which can lead to the reaction mass gelation [16], either leading to a decrease in the yield of the main product, or complete impossibility of one-step synthesis of benzoxazines based on diamines.

**Figure 2:**
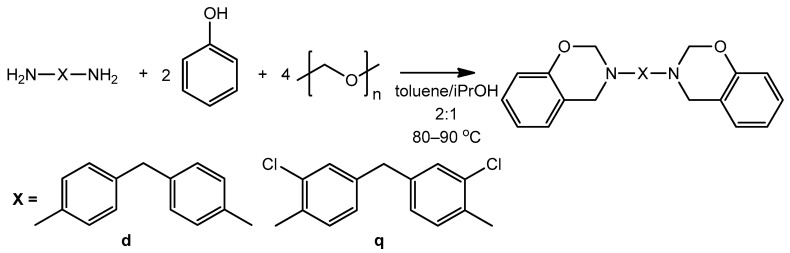
Synthesis of benzoxazine monomers based on diamines.

Despite the aforementioned fact, benzoxazines based on 4,4′-diaminodiphenylmethane/quamine, phenol and paraformaldehyde were obtained successfully (Figure 2) by two methods: in toluene and in a mixture of toluene and isopropanol according to the scheme below (Figure 2). The structure of the obtained benzoxazine monomers was characterized using ^1^H and ^13^C NMR spectroscopy.

**Figure 3:**
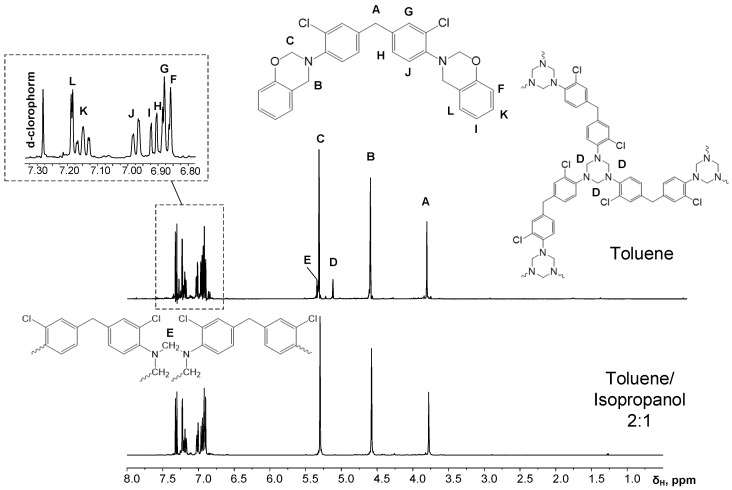
^1^H NMR spectrum of benzoxazine based on quamine in various solvents.

A low-intensity signal with a chemical shift at δ_H_ = 5.1 ppm in the ^1^H NMR spectra of the products obtained via this method (Figure 3, Table 2) indicates a small number of triazine structures.

**Figure 4:**
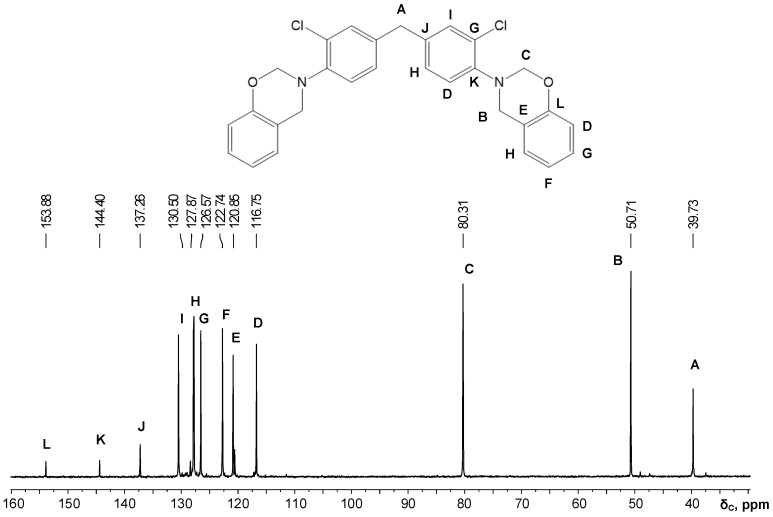
^13^C NMR spectrum of benzoxazine based on quamine in toluene/isopropanol 2:1.

In the second method, a toluene/isopropanol mixture with 2:1 volume ratio was used. Due to the hydroxyl groups affinity to isopropanol and increased general solvation, triazine structures are not formed (Figure 3 and Figure 4, Table 2) [35].

The yields of benzoxazines in the two preparation methods were up to 90–95%. To exclude the formation of compounds with the Mannich bridge, a 10% excess of paraformaldehyde was used, its loading was carried out gradually and only after complete dissolution of quamine and phenol, the optimal reaction temperature was used (80–90 °C).

Benzoxazine monomer based on 4,4′-diaminodiphenylmethane (P-d) was obtained by the analogous method in toluene/isopropanol solvents mixture.

Based on the data obtained from ^1^H NMR spectroscopy, it can be concluded that some branched triazine structures insoluble in toluene are formed. Thus, carrying out the reaction in a toluene/isopropanol solvent mixture gives a much cleaner product than in pure toluene medium. However, unlike a number of halogen-free diamines [14,17,18,19,20,21], the direct synthesis of P-q from quamine, phenol and paraformaldehyde in toluene medium is still possible despite the presence of a slightly larger number of byproducts in comparison with the toluene/ethanol solvent mixture.

**Figure 5:**
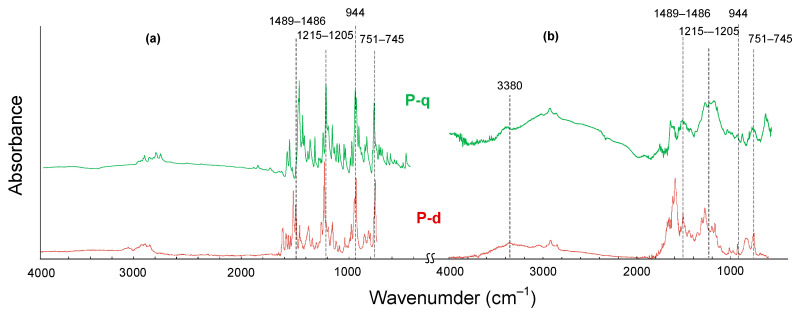
FTIR spectra of benzoxazine monomers (**a**) and polymers (**b**).

The structures of the obtained monomers and polymers were confirmed using FTIR spectroscopy and elemental analysis (Table 3). In the FTIR spectra of monomers (Figure 5), the absorption bands correspond to various vibrations in the oxazine ring: at 944 and 1215–1205 cm^−1^ –to the stretching vibrations of the C–N–C and C–O–C bonds, respectively; in the region of 1489–1486 cm^−1^ –to the stretching vibrations of the C–H bonds in the –CH_2_– groups; at 751–745 cm^−1^ –to the bending vibrations of –CH_2_– groups. In the FTIR spectra of polybenzoxazines, the intensities of these bands decrease significantly, and a broadened band at 3600–3380 cm^−1^ appears, corresponding to the stretching vibrations of phenolic hydroxyl groups linked by hydrogen bonds. The absorption bands in the FTIR spectra of benzoxazine and related polybenzoxazines correspond well to the literature data [36,37,38,39].

The content of elements in benzoxazine monomers (Table 3), taking into account the measurement error, converges with the theoretical calculation, therefore, pure monomers were obtained.

The obtained benzoxazine monomers are polymerized by the thermal method with the formation of an insoluble thermosetting polymer according to the scheme shown in Figure 8 (Figure S3 in Supplementary Materials).

### 3.2. Properties of Diamine-Based Benzoxazine Monomers

The curing kinetics of various diamine-based benzoxazine monomers was investigated by using differential scanning calorimetry (DSC) (Figure 8 and Figure S3, Table 4). The curves show that P-d polymerization proceeds under milder conditions than P-q. This may be due to the fact that the aromatic rings of the diamine in P-q are deactivated by chlorine atoms; therefore, the addition to the ortho position relative to the nitrogen atom will be somewhat difficult both sterically and energetically.

**Figure 6:**
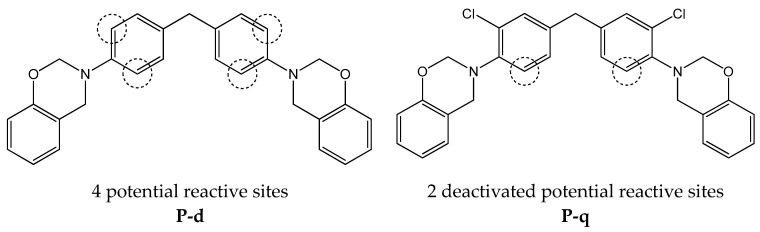
Possible reactive sites in the aromatic ring of the diamines and influence of substituents.

This statement is in a good agreement with early studies by Ishida and colleagues [40,41], who showed that benzoxazine monomers based on bisphenol A and aromatic amines polymerize not only according to the standard scheme with the formation of phenolic polymers with a Mannich bridge, but also with the participation of the p-position of the aromatic amine residue. The structure of the aromatic amine is important here. Amines with an activated aromatic ring (that is, with electron-donor substituents that increase the electron density in the o- and p-positions relative to the amino group) significantly increase the network density, while deactivated amines with a methyl group in the o- and p- positions, on the contrary, noticeably reduce it (Figure 6, Figure 8 and Figure S3) [42]. In other words, if benzoxazine monomer has an electron-donor substituent in the m-position relative to the nitrogen atom or has no substituents in the aromatic ring of the diamine, then polymerization proceeds under milder conditions (lower temperature of the onset of polymerization and lower enthalpy of the curing reaction) and higher cross-link density and glass transition temperature are achieved in the resulting polymer (Figure 14).

In this case the P-q monomer has an electron-withdrawing chlorine in o-position and as a result polyP-q has reduced T_g_ of 182 °C compared to polyP-d’s 190 °C. Studies [43,44] report that the presence of electro-acceptor groups in amine or phenol aromatic ring can significantly affect the conditions for obtaining the benzoxazine monomer, and also can increase the polymerization temperature, which is observed during the curing of P-q, too.

**Figure 7:**
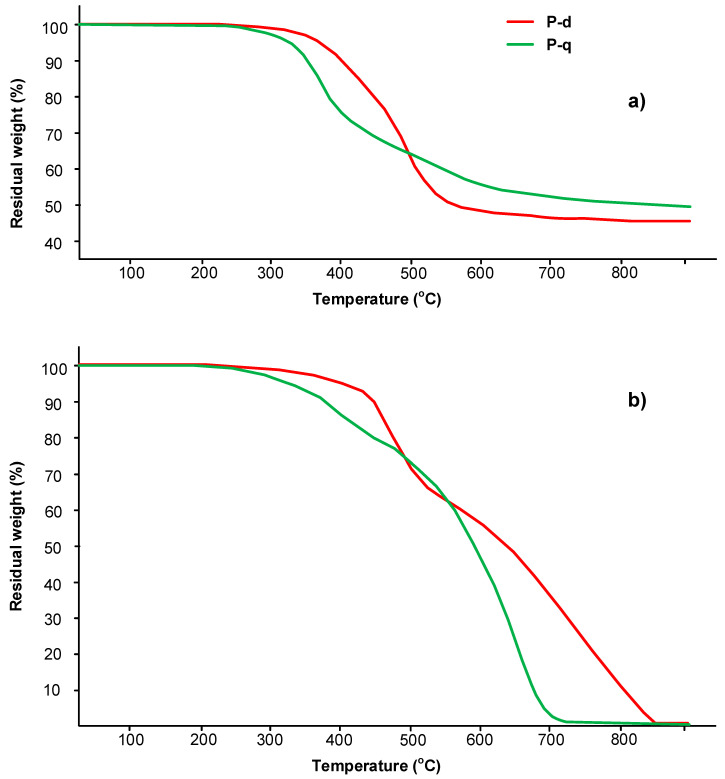
TGA curves of benzoxazine monomers P-d and P-q: (**a**) in the argon; (**b**) in the air atmosphere.

To confirm the stability of the benzoxazine monomers during curing in the selected mode, a thermogravimetric analysis both in air and argon atmosphere was carried out (Figure 7).

**Figure 8:**
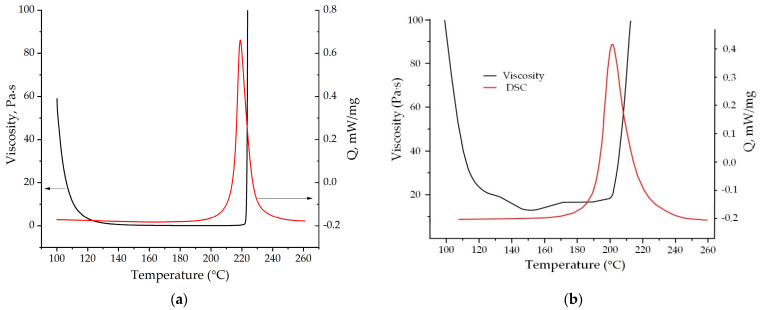
The comparison of viscosity (black) and DSC (red) curves of P-q (**a**) and P-d (**b**) at a heating rate 2 °C/min.

**Figure 9:**
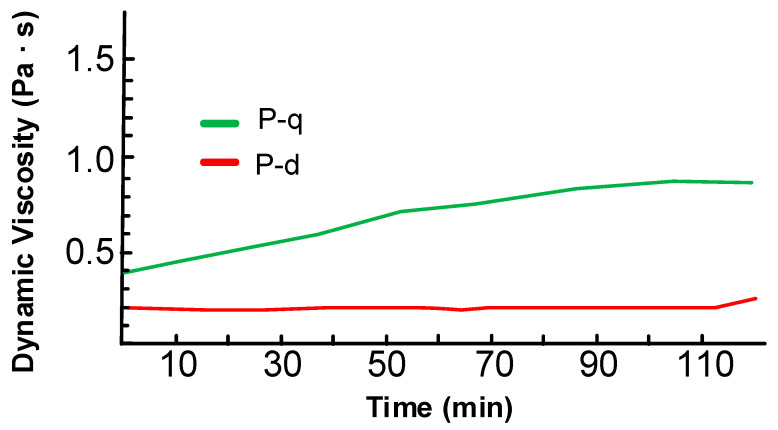
Change in the viscosity of benzoxazine monomers at 130 °C.

Figure 8 and Figure 9 show that the temperature of 5% mass loss for monomers P-d and P-q in air 407 °C and 333 °C, respectively, and in argon, 366 °C and 327 °C, respectively. These temperatures indicate the stability of the materials under the selected curing mode. The char yield at 800 °C for P-d and P-q in argon was 45 and 50%, respectively; in air, both monomers burned out without residue at 800 °C.

To determine the optimal curing mode of the obtained P-q, as well as the possibility of using it in the production of polymer composite materials and the processing method, we measured temperature profile of viscosity at a heating rate of 2 °C/min (Figure 8). It can be seen that the obtained benzoxazine monomer has a rather wide technological window (115–225 °C) at a low viscosity (<1 Pa·s), which makes it possible to process this monomer by the vacuum infusion method.

Figure 8 represents the comparison of the viscosity and DSC curves at the same heating rate of 2 °C/min. The dramatic increase in viscosity that corresponds to the gelation is observed right after the peak of DSC curve.

To evaluate the melting stability of P-q, viscosity curves were obtained in rotational mode at 130 °C for 2 h at a shear rate 200 rpm. Figure 9 shows that the P-q viscosity increase 2 times within 2 h; however, its values still remain below 1 Pa∙s. While viscosity of P-d monomer was not altered in a distinguishable way. This aspect contributes to the possibility of the vacuum infusion processing of monomers having P-d base more preferred than P-q.

### 3.3. Thermal Properties and Fire Resistance of Diamine-Based Polybenzoxazines

**Figure 10:**
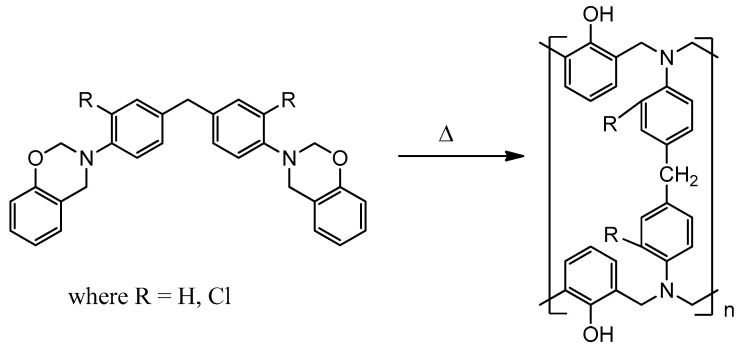
Scheme of polymerization of benzoxazine monomers.

Benzoxazine monomers were cured according to the following regime: 2 h at 180 °C, 4 h at 200 °C, 2 h at 220 °C; all samples were degassed at 130 °C for 1 h before curing. The polymerization scheme is shown in Figure 10. The oxazine rings are opened to form a crosslinked polymer under the high temperature.

Elemental analysis of the obtained polymers (Table 5) showed that the only target polymerization reaction takes place during polymerization; that is to say, destruction does not occur with the release of byproducts.

**Figure 11:**
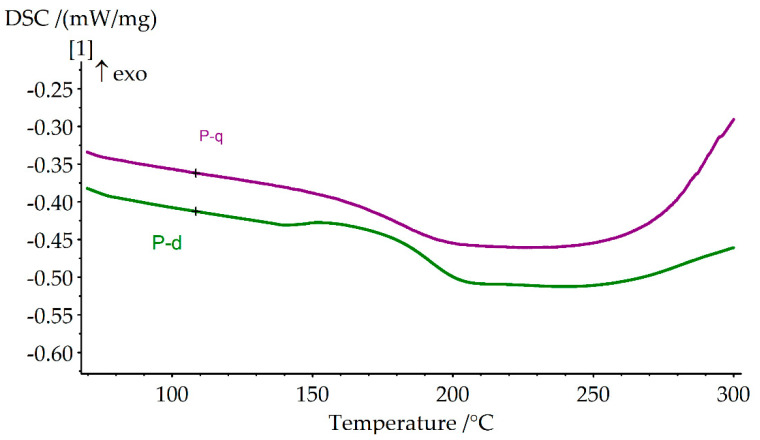
DSC curves of the cured P-q and P-d.

The glass transition temperatures (T_g_) of the obtained cured samples were measured by DSC at a heating rate 10 °C/min. It turned out that T_g_ for P-d is higher than for P-q, being 190 °C and 182 °C, respectively (Figure 11). By the character of DSC curves we could define that the beginnings of the thermal destruction of the P-q and P-d polymers are very close to each other in the region of 250 °C. However, the dramatic rise of DSC heat flow of P-q indicates that thermal stability of P-q at temperatures 250–300 °C is lower than that of P-d.

**Figure 12:**
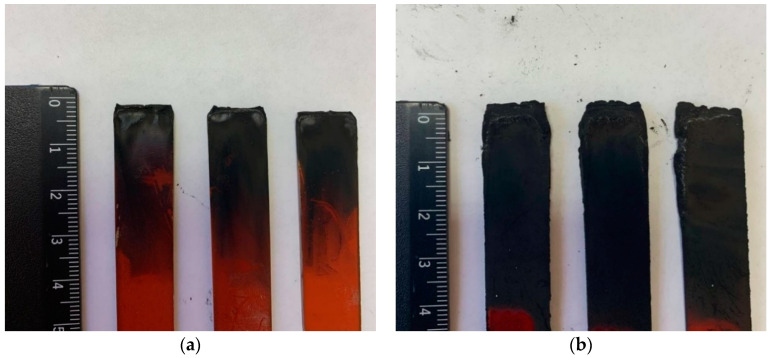
Photos of the tested samples of polyP-q (**a**) and polyP-d (**b**).

The determination of the fire-resistance according to the UL-94 standard showed that polyP-d has a category V-1, while polyP-q has an increased resistance to burning and so can be classified in the V-0 category (Table 6). In comparison with polyP-d, the quamine-based polymer is characterized by near-zero burning times, even after several flame applications. The examples of the tested samples are presented in Figure 12. The multicellular foam on the surface of the polyP-q samples seems to an the insulating effect resulting in the limitation of the diffusion of oxygen to the surface of the underlying material. The obtained flammability experimental data are in good agreement with the calculated data on the limiting oxygen index (LOI) according to the Van Crevelen–Hovtyzer rule [45] (1):

LOI = 17.5 + 0.4⋯CY(1)

where CY is the coke yield according to the TGA data.

One of the limiting factors of plastics’ high-temperature usage is their tendency not only to soften, but also to undergo the thermally induced degradation. Thermal degradation is the upper limit of the polymer’s operating temperature; above this temperature polymers can degrade with the formation of low-molecular-weight products that can change their properties.

The study of polybenzoxazines’ decomposition allows us to understand nature of the thermal stability of the material and prompt us to create new structures with greater thermal resistance.

It was proposed that the thermal decomposition of polybenzoxazines occurs stepwise [46,47,48]. At the first stage of the destruction, aromatic compounds are formed (benzene, derivatives of phenol, aniline). In the second step—low-molecular compounds (hydrocarbons, carbon dioxide, aliphatic amines, etc.), followed by carbonization.

**Figure 13:**
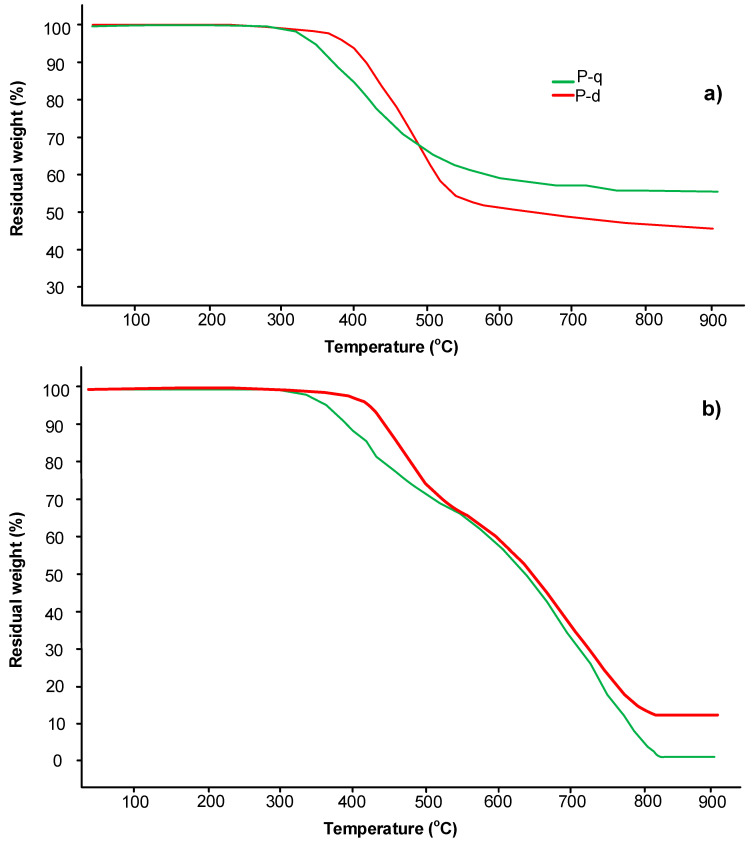
TGA curves of polyP-d and polyP-q: (**a**) in the argon; (**b**) in the air atmosphere.

In the present work, thermogravimetric analysis with a mass-detector was carried out. According to the TGA data (Figure 13 and Table 6), the 5% and 10% mass-loss of polyP-q occurs at lower temperatures compared to polyP-d. However, probably due to the presence of chlorine atoms, polyP-q has a higher char yield.

The structures formed during thermal destruction of polyP-q are described below (Table 7).

The result obtained is generally in satisfactory agreement with previously published studies. It can be noted that, both in air and in an inert atmosphere in the range of 300–500 °C, the destruction of benzoxazine cycles occurs predominantly and a small amount of potentially toxic chlorine-containing substances is released. As can be seen from Table 5, at low temperatures from 343 °C and 700 °C in air, compounds are formed containing chlorine: HClO and CH_3_Cl are formed with fractions 0.04% and 0.11%, respectively. More compounds containing chlorine are formed in an argon atmosphere at different temperatures (373–445 °C). For example, CH_3_Cl (26.86%), ^+^CH_2_CH_2_Cl (3.93%), CH_3_CH_2_Cl (4.59%), CH_3_OCl (5.5%), ^+^CH_2_CH_2_NH_3_Cl (4.59%). Above 500 °C, there is almost no mass loss in the inert atmosphere. In air, thermo-oxidative destruction is most intense at 700 °C.

**Figure 14:**
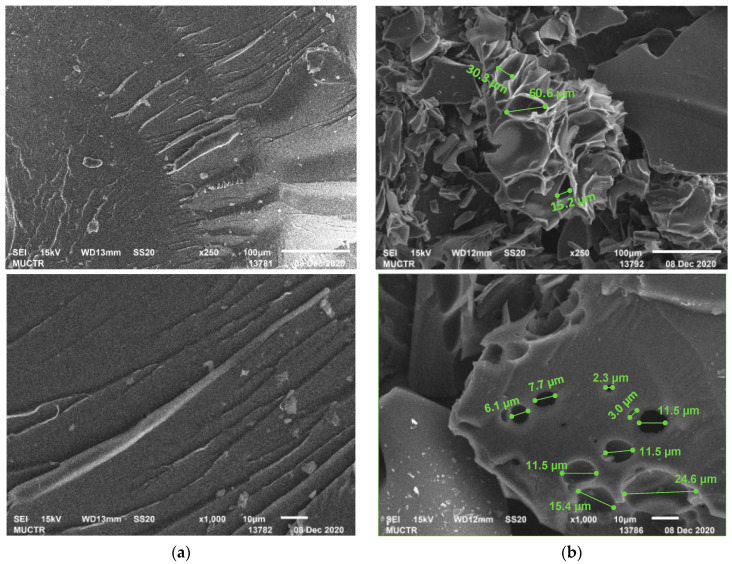
SEM analysis of polybenzoxazine polyP-q (**a**) and char yield (**b**) surfaces.

The surface of the fractured polymer sample and char yield were examined using SEM. Figure 14a shows that the polymer has glassy bulky surface with no specific morphological features. The SEM image of the char residue (Figure 14b) showed that during the burning of polybenzoxazine, a dense foam protective layer with a pore diameter from 2.3 to 60.6 µm is formed, which prevents the polymer from further burning.

It is also known that halogen radicals compete for the radical species HO· and H· that are critical for flame propagation [49,50]. Thus, the higher fire resistance of polyP-q compared to polyP-d can be explained by two factors. The first factor is the release of low molecular weight products, such as CO_2_, NO_2_, CH_3_Cl and HClO, which cause system cooling and removing of high-energy HO· and H· radicals. The second factor is the formation of the char layer on the polymer surface, which prevents oxygen penetration into the bulk of the sample and thereby stops combustion.

The notable feature of polyP-q is its higher thermal resistance in air, compared to the results in argon (Figure 13). This behavior usually occurs when thermo-oxidative reactions with the participation of atmospheric oxygen occur additionally in air, leading to the formation of densely crosslinked products. This is also confirmed by the results of TGA-MS analysis (Table 7). In an oxygen atmosphere at a temperature of about 340 °C, active particles (cations) are formed, which can additionally cross-link the polymer network. In an argon atmosphere, these particles are also formed, but already at higher temperatures. The formation of a char layer and small pores can be observed in the SEM images of the samples tested according to the UL-94 standard and the surface of the char residue examined using SEM (Figure 12 and Figure 14).

**Figure 15:**
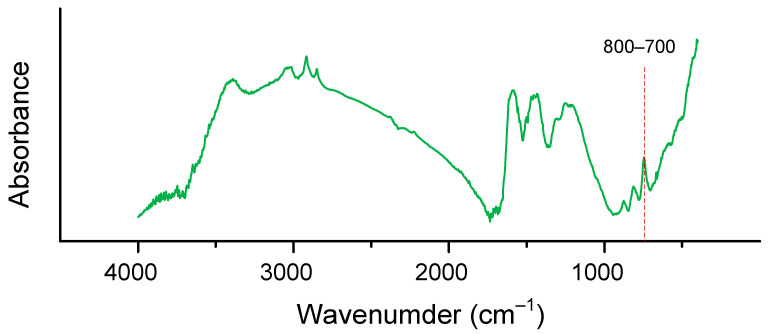
FTIR spectrum of the char residue of P-q-based polybenzoxazine.

Quamine-based polybenzoxazine char residue was investigated by FTIR (Figure 15). It showed stretching vibrations of the C–Cl (800–700 cm^−1^) bond after burning of the polymer sample. This observation is consistent with relatively low content of chlorine-containing ions during TGA and with the formation of a dense char residue in the UL-94 vertical test and presumably indicates the cessation of combustion on the sample surface without the flame penetrating deep into the sample.

Based on the data of elemental analysis and IR spectroscopy of the coke residue (Table 5, Figure 15), it can be seen that the residue contains a small amount of chlorine atoms. According to the TGA-MS data in air (Table 7), it can be seen that only 0.11% and 0.04% of the chlorine-containing products CH_3_Cl and HClO are formed, respectively. The data obtained indicate a low lability of chlorine atoms in the composition of polybenzoxazine, which is a positive factor in terms of safety and environmental friendliness.

The lower temperature of the onset of polyP-q decomposition in an inert atmosphere is most likely due to the easier cleavage of bonds in the polymer due to the presence of electron-withdrawing chlorine atoms in the diamine structure. On the contrary, the higher thermal resistance of polyP-q in air indicates a greater proportion of crosslinking and resulted char formation. This fact is indirectly confirmed by the presence of a significant amount of residual chlorine in the char residue, as well as a relatively low chlorine content in the combustion products.

Thus, the formation of char probably plays a major role in the mechanism of combustion and thermooxidative destruction of polyP-q.

## 4. Conclusions

A new, previously not reported in the literature benzoxazine monomer based on 3,3′-dichloro-4,4′-diaminodiphenylmethane (quamine) and phenol (P-q), was synthesized in a high yield (90–95%). P-q has a fairly wide technological window with a viscosity of less than 1 Pa·s at a temperature range of 120–200 °C, so it can be used as a binder in the production of polymer composite materials by vacuum infusion or RTM. Compared to the well-known commercial benzoxazine monomer P-d (obtained from phenol and 4,4′-diaminodiphenylmethane), this new polymer has a higher char yield (57% for polyP-q and 46% for polyP-d at 800 °C) and UL-94 V-0 fire resistance rating. The obtained results indicate the possibility to applicate P-q as a co-monomer in the production of fire-resistant binders for polymer composite materials, except for areas where the use of halogen-containing flame-retardants is not allowed.

## Figures and Tables

**Table 1:** The amounts of starting reagents for the synthesis of benzoxazine monomers based on diamines of various structures in various solvents.

Reagent		Benzoxazine Monomer		
		P-q		P-d
		Toluene	Toluene/Isopropanol = 2:1	Toluene/Isopropanol = 2:1
Diamine	g	26.715	20	20
	mol	0.100	0.075	0.100
Phenol	g	18.822	14.091	18.99
	mol	0.200	0.150	0.200
Paraformaldehyde, 91% (10% Excess)	g	14.52	10.87	14.65
	mol	0.440	0.330	0.440
Solvent	mL	150	150	150

**Table 2:** ^1^H and ^13^C NMR spectroscopy data for benzoxazine monomers.

Sample	Proton Chemical Shifts Δ_h_ (ppm)				Carbon Chemical Shifts Δ_c_ (ppm)			
	Oxazine Ring		Diamine		Oxazine Ring		Diamine	
	CH_2_N	CH_2_O	CH_2_	CH (Ar)	CH_2_N	CH_2_O	CH_2_	CH (Ar)
P-d	4.70	5.44	3.96	6.96–7.49	50.15	79.39	40.01	116.69–154.17
P-q	4.58	5.30	3.78	6.81–7.35	50.71	80.31	39.73	116.75–153.88

**Table 3:** Elemental analysis data for benzoxazine monomers.

Elements	Calculated, %	Found, %
P-d		
**C**	80.16	80.03
**H**	6.03	6.07
**O**	7.36	7.61 *
**N**	6.45	6.29
**P-q**		
**C**	69.19	68.86
**H**	4.81	4.62
**O**	6.36	6.52 *
**N**	5.56	5.66
**Cl**	14.08	14.34

* These values are obtained by difference.

**Table 4:** Differential scanning calorimetry results.

Parameter		P-d	P-q
**Temperature Characteristics of Curing Exotherm (°C)**	**Onset**	224	242
	**Peak**	234	247
	**End**	246	253
**Polymerization Heat (J/g)**		310	295

**Table 5:** Elemental analysis data for polybenzoxazines and char residue.

Elements	Calculated, %		Found, %			
			By Elemental Analysis		Surface X-ray Spectrometry ^1^	
	Polymer	Char	Polymer	Char	Polymer	Char
**P-d**						
**C**	80.16	-	79.97	87.78	78.68	91.94
**H**	6.03	-	5.99	2.05	-	-
**O**	7.36	-	7.79 ^2^	4.41 ^2^	21.32	8.06
**N**	6.45	-	6.25	5.76	-	-
**P-q**						
**C**	69.19	-	68.52	89.30	73.53	91.81
**H**	4.81	-	4.80	1.59	-	-
**O**	6.36	-	6.64 ^2^	2.77 ^2^	23.83	7.64
**N**	5.56	-	5.63	4.87	-	-
**Cl**	14.08	-	14.41	1.47	2.64	0.56

^1^ Measurement was carried out using scanning-electron microscope (Jeol JSM-U3, Tokyo, Japan) with energy dispersive X-ray spectrometer (Eumex, Heidenrod, Germany) in accelerating voltage of 15 kV. ^2^ These values are obtained by difference.

**Table 6:** Thermal properties of benzoxazines based on diamines.

Parameter		PolyP-d	PolyP-q
**T_g_ (°C) ^a^**		190	182
**UL-94**	**τ_1_, s**	18	5
	**τ_2_, s**	10	0
	**τ_3_, s**	7	2
	**τ_4_, s**	20	1
	**τ_5_, s**	5	0
	**τ_tot_, s**	60	8
	**Afterglow, s**	No	No
	**Particles or Drops**	No	No
	**Flammability Rating**	V-1	V-0
**LOI ^b^**		36	40
**TGA in Argon**			
**T_5%_ (°C)**		395	357
**T_10%_ (°C)**		416	380
**W_800_ (%)**		46	57
**W_900_ (%)**		45	57
**TGA in Air**			
**T_5%_ (°C)**		423	375
**T_10%_ (°C)**		445	395
**W_800_ (%)**		14	5
**W_900_ (%)**		12	1

^a^ Measured by DSC (Supplementary Materials). ^b^ Calculated by Van Krevelen–Hovtyzer equation (char yield at 800 °C values were used for calculation) [45].

**Table 7:** The results of TG-MS analysis of volatile products of polyP-q in air and inert atmosphere.

№	Probable Structure	*m*/*z* Calculated	*m*/*z* Observed	Intensity × 10^12^	Fraction ^a^, %	T, °C
Air						
1	^+^CH_2_–N–CH_2_^+^	43.04	42.3	24	10.52	343
2	^+^CH_2_N = CH_2_	42.03				
3	CH_3_N = CH_2_	43.04				
4	CH_3_Cl	49.99	50.4	0.25	0.11	
5	HClO	51.97	52.3	0.1	0.04	
6	CO_2_	43.99	44.2	200	87.66	700
7	CH_3_CH_2_NH_2_	45.06	45.3	2.8	1.23	
8	NO_2_	45.99	46.2	1	0.44	
**Argon**						
9	CO_2_	43.99	44.2	5.8	18.87	373
				8.6		445
10	CH_3_Cl	49.99	50.2	3.0	26.86	373
				3.2		438
				3.9		445
			51.3	3.0		373
				3.4		438
				4.0		445
11	^+^CH = CH_2_CN	52.02	52.3	2.9	12.19	373
				3.3		438
				3.1		445
12	^+^CH_2_CH_2_Cl	63.0	63.3	3.0	3.93	373
						438
						445
13	CH_3_CH_2_Cl	64.01	65.3	3.5	4.59	438
14	CH_3_OCl	65.99		4.2	5.50	445
15	^+^CH_2_NH_3_Cl	66.02	66.8	3.5	4.59	445
16	HOCH_2_NHCH_2_OH	77.05	77.4	3.5	4.59	438
17	C_6_H_5_^+^	77.04				445
18	C_6_H_6_	78.05	78.4	3.6	4.72	438
19	CH_3_CH_2_N = NCH_2_CH_3_	86.08	85.4	2.6	3.41	373
20	C_6_H_5_NH_2_	93.06	93.3	2.8	3.67	438
21	C_6_H_5_CH_3_	92.06				445
22	C_6_H_5_OH	94.04	95.5	2.5	3.28	373
23	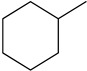	98.11	98.1	2.9	3.80	445

^a^ Volatile product fraction is equal to the ratio of the intensity of the given substance to the sum of all intensities at whole temperature range.

## Data Availability

The data presented in this study are available on request from the corresponding author.

## Supplementary Materials

The following are available online at https://www.mdpi.com/article/10.3390/polym13091421/s1, Figure S1: ^1^H NMR spectrum of benzoxazine based on 4,4′-diaminodiphenylmethane in toluene/isopropanol 2:1, Figure S2: ^13^C NMR spectrum of benzoxazine based on 4,4′-diaminodiphenylmethane in toluene/isopropanol 2:1, Figure S3: The DSC curves describing the curing process of diamines-based benzoxazines (heating rate 10 deg/min), Figure S4: DSC curves of polybenzoxazines based on diamines (heating rate 10 deg/min), Figure S5: Mass spectrum of degradation of polybenzoxazine P-q in air at 343 °C, Figure S6: Mass spectrum of degradation of polybenzoxazine P-q in argon at 373 °C, Figure S7: Mass spectrum of degradation of polybenzoxazine P-q in argon at 438 °C, Figure S8: Mass spectrum of degradation of polybenzoxazine P-q in argon at 445 °C.
