# Supplementary material for: Benzoxazine Monomers and Polymers Based on 3,3′-Dichloro-4,4′-Diaminodiphenylmethane: Synthesis and Characterization
Source: Polymers (Basel). 2021 Apr 28;13(9):1421. doi: 10.3390/polym13091421 (PMC8125557; doi:10.3390/polym13091421)
Supplement: Supplementary file 1 [file polymers-13-01421-s001.zip › polymers-1064227-supplementary.pdf]

polymers

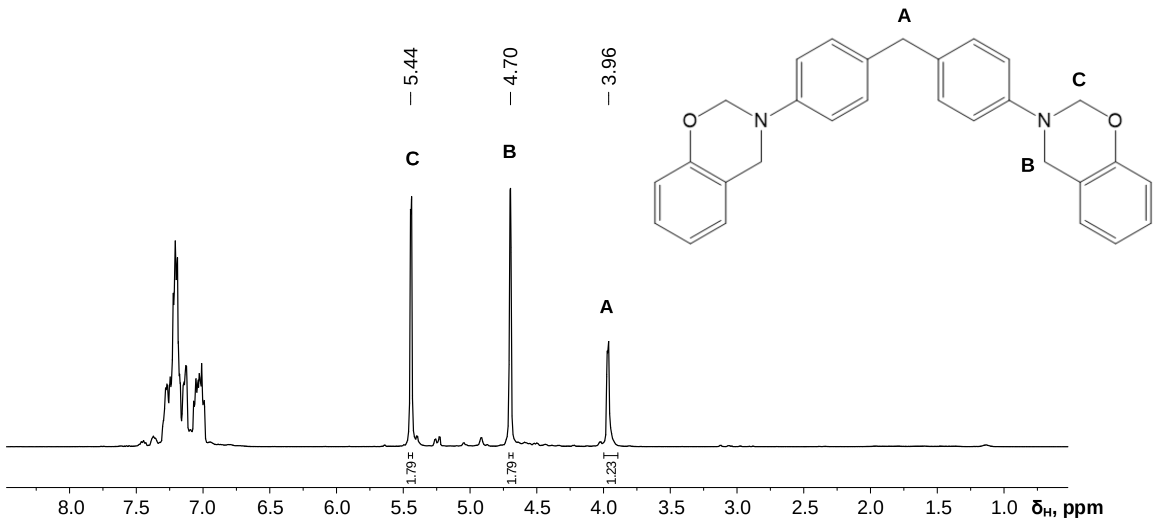

Figure S1. ^{1}H NMR spectrum of benzoxazine based on 4,4′-diaminodiphenylmethane in toluene/isopropanol 2:1.

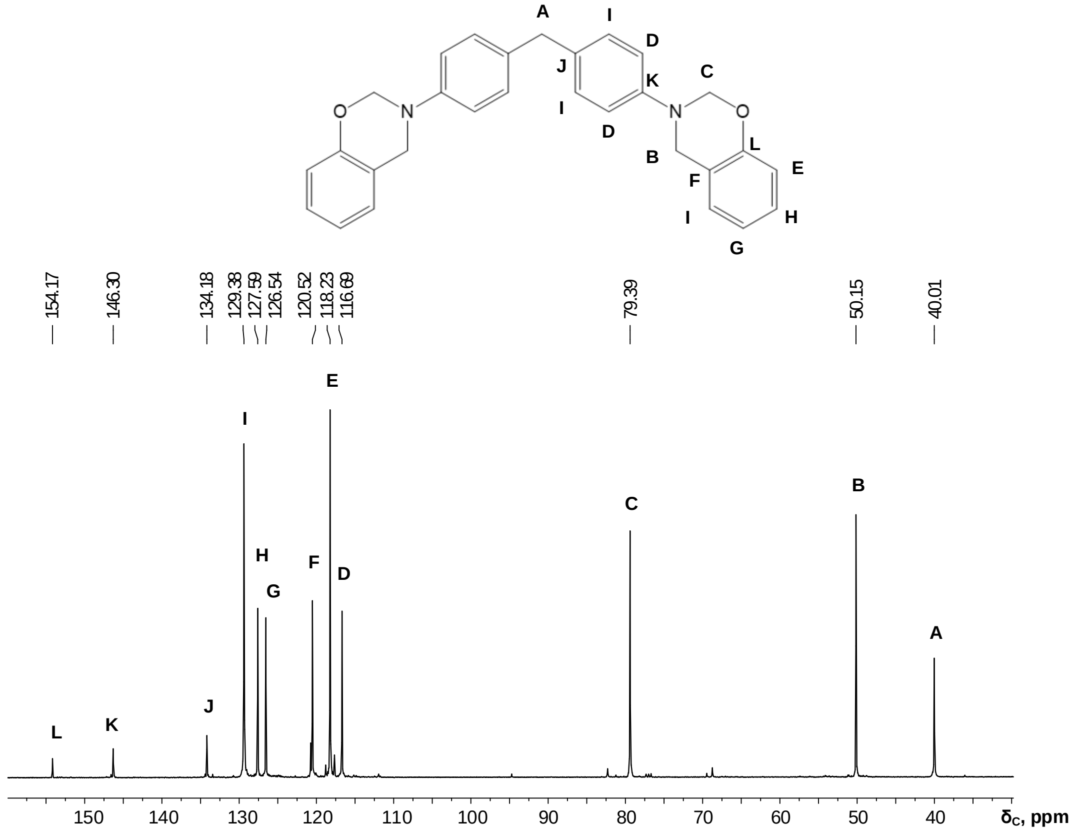

Figure S2. ^{13}C NMR spectrum of benzoxazine based on 4,4′-diaminodiphenylmethane in toluene/isopropanol 2:1.

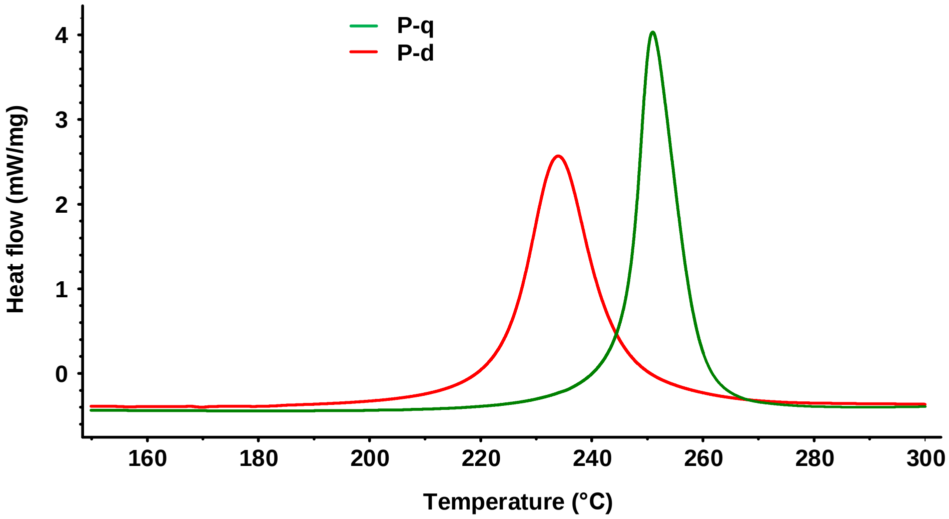

Figure S3. The DSC curves describing the curing process of diamines-based benzoxazines (heating rate 10 deg / min).

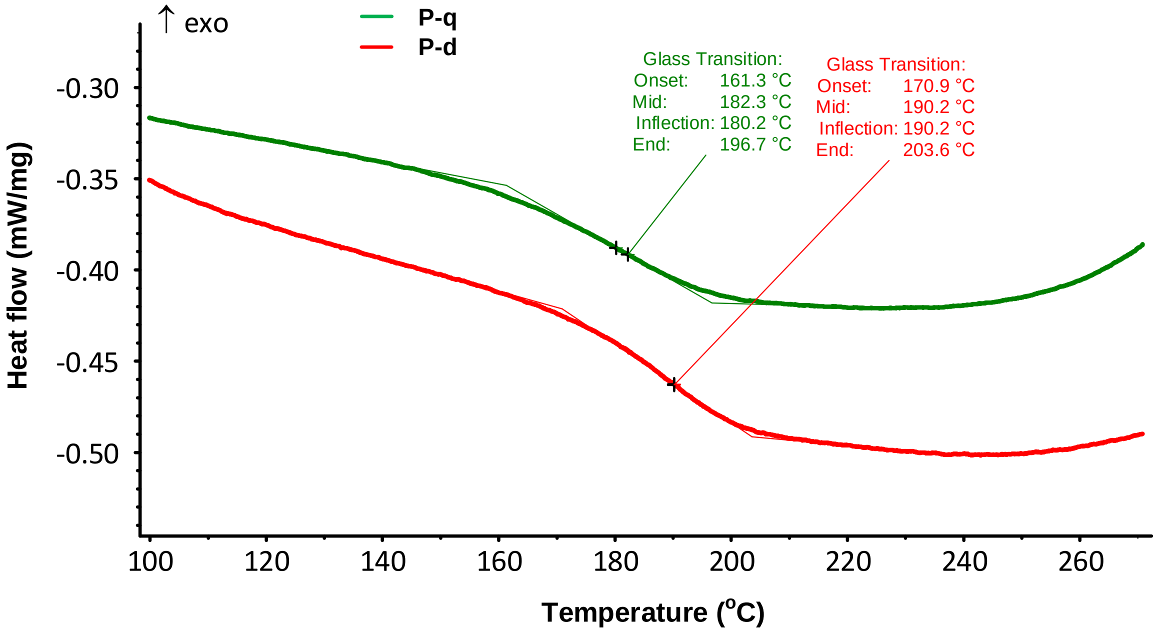

Figure S4. DSC curves of polybenzoxazines based on diamines (heating rate 10 deg / min).

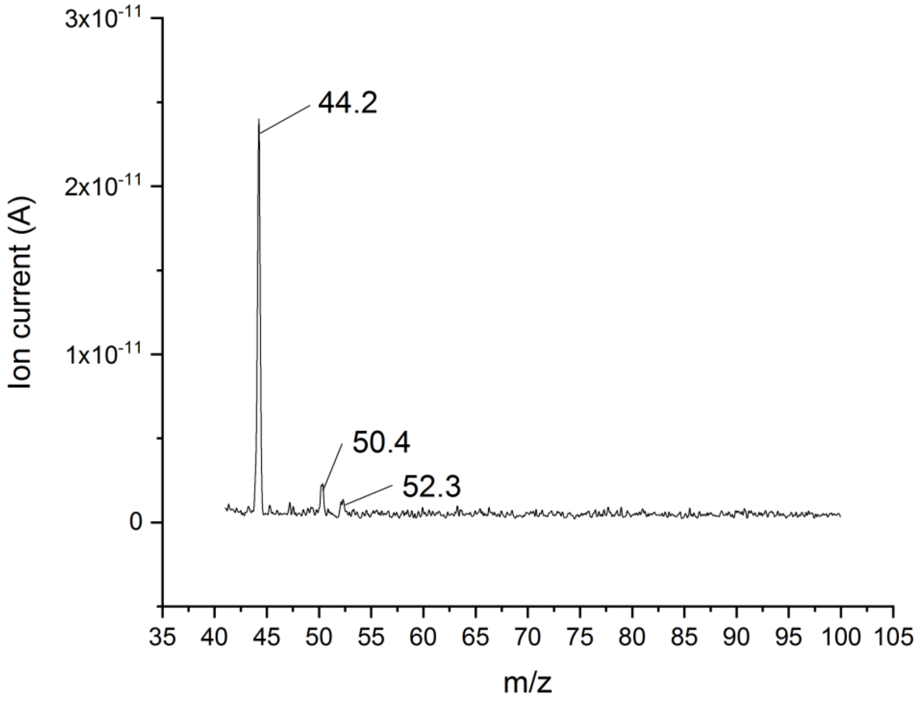

Figure S5. Mass spectrum of degradation of polybenzoxazine P-q in air at 343 °C.

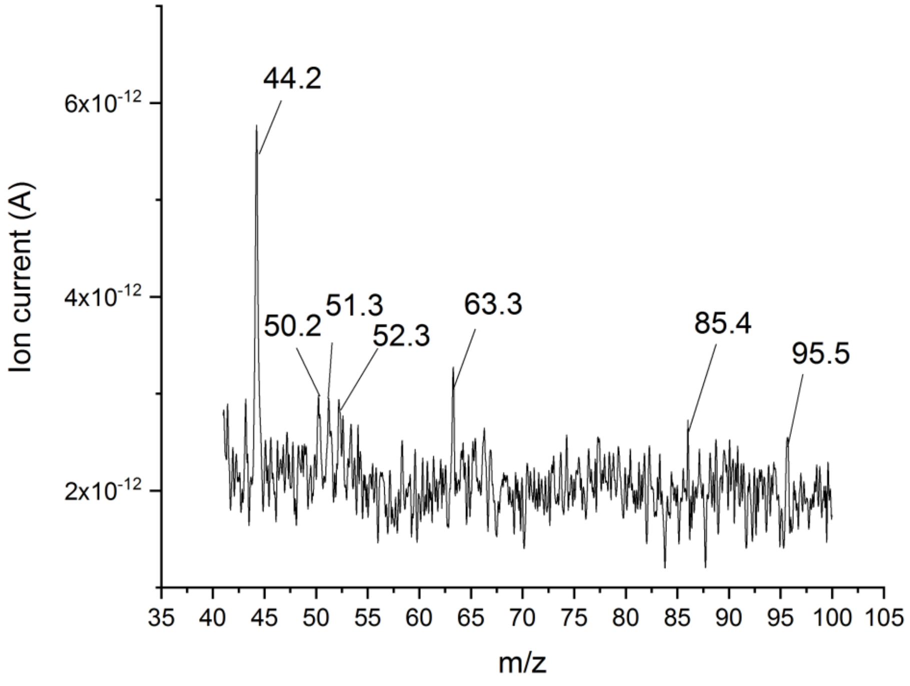

Figure S6. Mass spectrum of degradation of polybenzoxazine P-q in argon at 373 °C.

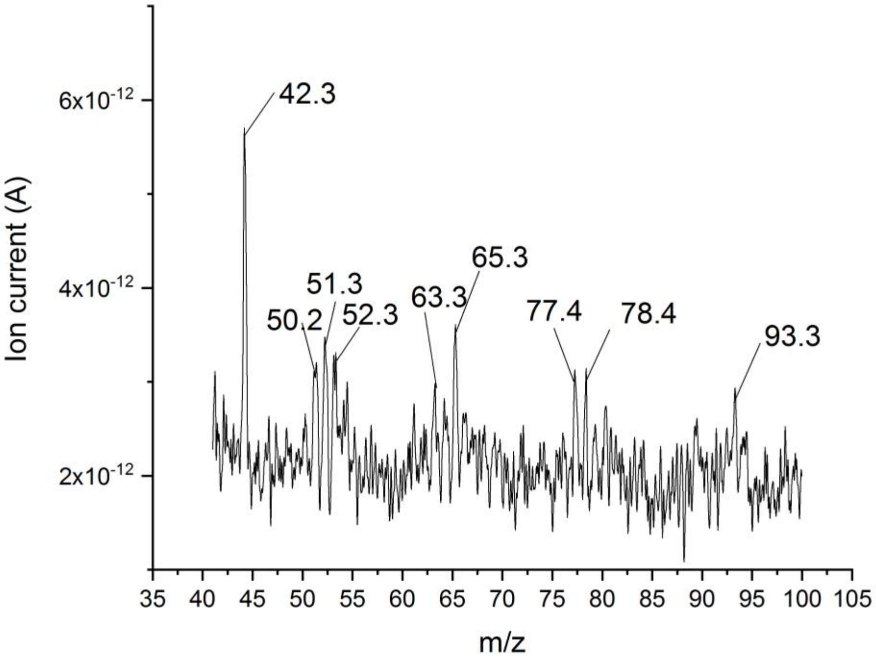

Figure S7. Mass spectrum of degradation of polybenzoxazine P-q in argon at 438 °C.

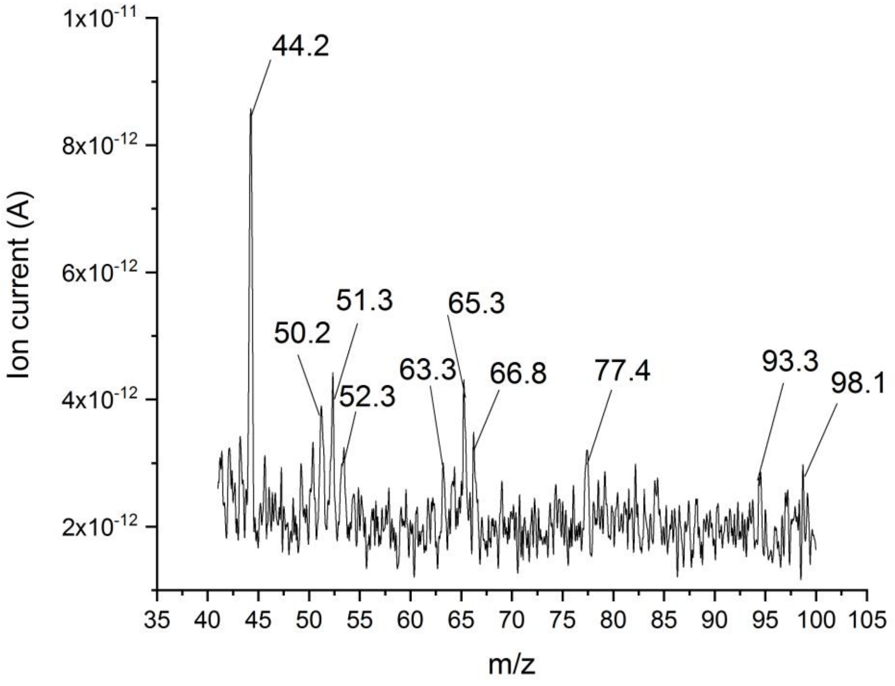

Figure S8. Mass spectrum of degradation of polybenzoxazine P-q in argon at 445 °C.
